# *Niemann - Pick C2* regulates steroid hormone secretion and lipid deposition in chicken follicular granulosa cells

**DOI:** 10.1016/j.psj.2025.105340

**Published:** 2025-05-26

**Authors:** Ruihao Yu, Shuo Wei, Felix Kwame Amevor, Liuting Wu, Dan Xu, Zheliang Liu, Kunlong Qi, Yingjie Wang, Gang Shu, Xiaoling Zhao

**Affiliations:** a State Key Laboratory of Swine and Poultry Breeding Industry, College of Animal Science and Technology, Sichuan Agricultural University, Chengdu, Sichuan, PR China; b Farm Animal Genetic Resources Exploration and Innovation Key Laboratory of Sichuan Province, College of Animal Science and Technology, Sichuan Agricultural University, Chengdu, Sichuan, PR China; c Key Laboratory of Livestock and Poultry Multi-omics, Ministry of Agriculture and Rural Affairs, Sichuan Agricultural University, Chengdu, Sichuan, PR China; d Department of Basic Veterinary Medicine, Sichuan Agricultural University, Chengdu, Sichuan, PR China

**Keywords:** Chicken, follicular granulosa cell, Lipid, Steroid hormone, *Niemann - Pick C2*

## Abstract

Follicular development is tightly regulated by the coordinated action of multiple hormones and complex gene regulatory networks in granulosa cells, which play a crucial role in egg production and fertility in hens. Extensive studies have established that *Niemann-Pick C2* (*NPC2*) is a key regulator of cholesterol metabolism and steroid hormone secretion in mammals. However, its specific role in chicken ovarian granulosa cells remains unclear. In this study, cultured chicken ovarian granulosa cells were used to investigate the function of *NPC2* through transfection with *NPC2* overexpression vectors or small interfering RNAs (siRNAs). The results showed that silencing *NPC2* significantly increased the expression of *SREBP1, SREBP2, LPL, SCD1, CPT1* and *DGAT2* genes involved in lipid synthesis (*P* < 0.01), and also increased the synthesis of Triglyceride (TG) and Cholesterol (TC) in granulosa cells (*P* < 0.05), whereas *NPC2* overexpression led to a marked reduction in the expression of these indicators of lipid metabolism (*P* < 0.01). Furthermore, *NPC2* knockdown significantly inhibited the production of progesterone (P_4_) (*P* < 0.01) and estradiol (E_2_) (*P* < 0.05), along with the expression of *STAR, CYP17A1* steroidogenesis-related genes in granulosa cells (*P* < 0.05). Conversely, *NPC2* overexpression enhanced P_4_ and E_2_ synthesis and upregulated the expression of genes associated with steroid hormone biosynthesis (*P* < 0.05). Taken together, these findings indicate that *NPC2* suppresses lipid accumulation while promoting steroid hormone production in chicken granulosa cells, which highlights its potential regulatory role in ovarian function.

## Introduction

The economic efficiency of commercial laying hens depends on their egg production capacity, which in turn requires efficient follicular development to sustain high egg yields (Tang et al., 2024). During sexual maturation in chickens, proper follicular development relies on interactions between oocytes and granulosa cells. As follicles mature, granulosa cells establish gap junctions with the oocyte, facilitating the exchange of nutrients and signaling molecules (Martinez et al., 2023). These interactions regulate transcriptional and post-transcriptional processes within the oocyte, playing a crucial role in the growth and maturation of primordial follicles (Navarro-Costa et al., 2005; Fiorentino et al., 2023). Oocytes also influence neurotransmitter secretion and immune responses by releasing bioactive factors that support granulosa cell development, promote their proliferation (Regassa et al., 2011), and prevent premature differentiation (Eppig, 2001; Matzuk et al., 2002; Sugiura et al., 2005; Fitzharris and Baltz, 2006). Moreover, follicular development in chickens is closely associated with lipid metabolism and the synthesis and secretion of steroid hormones. It has been shown that the oxidation and synthesis of fatty acids affect the proliferation of bovine GCs and steroid synthesis, which indirectly affect the development of bovine follicles (Elis et al., 2015). Furthermore, during follicular maturation, oocytes regulate follicle progression by secreting bone morphogenetic protein 15 (*BMP15*) and growth differentiation factor 9 (*GDF9*), which inhibit luteinizing hormone receptor (*LHR*) and follicle-stimulating hormone receptor (*FSHR*) expression. This suppression, in turn, reduces gonadotropin-induced progesterone production in granulosa cells (Otsuka, 2010).

*NPC2* has been extensively studied in mammalian lipid metabolism, particularly in cholesterol trafficking and steroid hormone biosynthesis (Rasmussen et al., 2023; Zhao et al., 2023). It specifically binds cholesterol and facilitates its transport from lysosomes to other cellular compartments. Functional deficiencies or mutations in *NPC2* impair this transport process, leading to disrupted cholesterol homeostasis and lysosomal storage disorders, such as Niemann-Pick disease type C2 (Rasmussen et al., 2023; Zhao et al., 2023).

In humans, *NPC2* mutations cause cholesterol and lipid accumulation in the brain, liver, and spleen, leading to neurodegenerative disorders and organ enlargement (Liu et al., 2008; Vanier, 2010; Wiweger et al., 2021). Similar findings have been reported in mice, thus, *NPC2*-deficient mice fed a lithogenic diet exhibited reduced biliary cholesterol secretion, while *NPC2*-transgenic mice with liver-specific overexpression showed accelerated cholesterol deposition and gallstone formation under the same dietary conditions (Acuña et al., 2016; Rasmussen et al., 2023). Cholesterol serves as a vital precursor for steroid hormone biosynthesis. Recent studies indicate that *NPC2* is essential for the production of oxysterols and steroid hormones by mediating cholesterol transport from late endosomes and lysosomes (LE/LYSs) to mitochondria, where steroidogenesis initiates (Juhl et al., 2021). These findings underscore the critical role of *NPC2* in regulating intracellular cholesterol trafficking and steroid hormone synthesis. *NPC2* is highly conserved among canines (Ellerbrock et al., 1994), bovines (Larsen et al., 1997), and chimpanzees (Fröhlich and Young, 1996), with more than 75 % sequence similarity among mammals and 55-70 % similarity in other vertebrates (Pelosi et al., 2014). This suggests that NPC2’s function is evolutionarily conserved, supporting the notion that it performs similar physiological roles across diverse species.

Given the essential role of *NPC2* in lipid metabolism and steroid hormone synthesis in mammals and the reproductive impairments associated with its dysfunction we hypothesize that *NPC2* may play a similar regulatory role in chicken granulosa cells. However, the molecular mechanisms through which *NPC2* modulates lipid homeostasis and steroidogenesis in chicken follicular granulosa cells remain largely unknown. unknown. Therefore, this study aims to elucidate the role of *NPC2* in regulating steroid hormone secretion and lipid accumulation in chicken granulosa cells, providing novel insights into its function during avian follicular development. To validate its function, we isolated primary follicular granulosa cells from chickens and conducted oil red O staining, quantitative real-time PCR (qRT-PCR), and Western blot analyses. A deeper understanding of the role of *NPC2* in chicken granulosa cells will provide insights into the molecular mechanisms governing follicular development and ovarian function as well as offer a theoretical foundation and practical guidance for improving egg-laying performance and reproductive efficiency in laying hens.

## Materials and methods

### Animals and sample collection

All experimental animal procedures received ethical approval from the Institutional Animal Care and Use Committee (Certification No.SYXK2019-187) at Sichuan Agricultural University, with experimental protocols adhering to the institution's Laboratory Animal Welfare and Ethics standards.

Follicular tissues were collected from healthy laying Rohman hens raised at the Poultry Breeding Unit of Sichuan Agricultural University (Ya'an, China). Granulosa cells (GCs) were isolated from the hierarchical follicles by carefully removing the yolk in phosphate-buffered saline (PBS, Hyclone, Logan, UT, USA). The granulosa layer was then separated from the theca layer, and the tissue was immediately frozen in liquid nitrogen before being stored at −80°C for subsequent RNA extraction and analysis.

### Cell culture and transfection

The isolation and culture of granulosa cells followed established protocols described in previous studies (Gilbert et al., 1977). Briefly, after humane euthanasia by cervical dislocation in accordance with animal welfare guidelines, the granulosa layer was dissected and finely minced. The tissue was then digested with 0.1 % type II collagenase (BaiTai Biotechnology, Chengdu, China), followed by filtration through a 70 μm cell strainer. The resulting cell suspension was resuspended in Dulbecco’s Modified Eagle Medium (DMEM, Gibco, Carlsbad, CA, USA) supplemented with 10 % fetal bovine serum (Gibco, Grand Island, NY, USA) and 0.1 % penicillin-streptomycin (Invitrogen, Carlsbad, CA, USA). The isolated GCs were incubated at 37°C in a humidified atmosphere with 5 % CO_₂_ for 3 h to allow cell attachment. The siRNA sequences used in this study are listed in Table 1. The primary granulosa cells were seeded in 6-well plates for transient transfection experiments. Non-adherent cells were removed by replacing the medium after initial attachment, and the adherent GCs were subsequently cultured under standard conditions with medium changes every 24 h. When the cells reached approximately 60-80 % confluence, they were transfected with either the pcDNA3.1-*NPC2* overexpression plasmid or siRNA-*NPC2* (2500 ng/well) using Lipofectamine 3000 reagent (Invitrogen, USA), according to the manufacturer’s instructions. Forty-eight hours post-transfection, the cells were harvested for RNA extraction.

**Table 1:** Primers used for qPCR.

Gene Name	Forward (5′−3′)	Reverse (5′−3′)
*NPC2*	*AAGACGGAAGCATTCAAGAGG*	*TCAGGTAGCTGTACGAGTGGC*
*NPC2-si-1*	*GAAGCAUUCAAGAGGUGAATT*	*UUCACCUCUUGAAUGCUUCTT*
*NPC2-si-2*	*CAGUAAGGCAAAGGUGUAUTT*	*AUACACCUUUGCCUUACUGTT*
*NPC2-si-3*	*CGUACAGCUACCUGAACAATT*	*UUGUUCAGGUAGCUGUACGTT*
*SREBP1*	*CGAGTACATCCGCTTCCTGC*	*TGAGGGACTTGCTCTTCTGC*
*SREBP2*	*GGACAGATGCCAAGATGC*	*GGTCAATGCCCTTCAACA*
*LPL*	*TGGACATTGGTGACCTGCTTATGC*	*TCGCCTGACTTCACTCTGACTCTC*
*SCD1*	*ACCTTAGGGCTCAATGCCAC*	*TCCCGTGGGTTGATGTTCTG*
*CPT1*	*ACAGGGCTTTGGGTTGC*	*CCTCATCATTCATAAGTGGC*
*DGAT2*	*AAGGGATTTGTGAAACTGGC*	*ACGTACACGCTGTGGTAGAAG*
*STAR*	*TGTTCCGCCTGGAGGTGGTGGTGGA*	*GGGAGCACCGAACACTCACAA*
*CYP17A1*	*CTTCAGGTGTTTCTCTTCCTCCTC*	*CTGTGGTTTCATGGCTGGATC*
*GAPDH*	*CGGATTTGGCCGTATTGG*	*GGTCACGCTCCTGGAAGAT*

### Plasmid construction

To investigate the role of the *NPC2* in GC, an empty pcDNA3.1 vector (named as NC—NPC2) was used as a control, while an *NPC2* overexpression plasmid (OE-NPC2) and siRNA targeting *NPC2* (si-NPC2) were synthesized by Tsingke Biotechnology (Tsingke, Beijing, China) and GenePharma (GenePharma, Shanghai, China), respectively.

### Oil red O staining

Lipid droplet accumulation was assessed using an Oil Red O staining kit according to the manufacturer’s protocol. Forty-eight hours post-transfection, cells were stained, and lipid droplet deposition in GCs was visualized using an electron microscope (DP80 Digital, Olympus, Tokyo, Japan). Images were captured at 400× magnification, and lipid droplet coverage was quantified using ImageJ software (National Institutes of Health, Bethesda, MD, USA) by measuring the relative area of lipid droplets compared to the cell nucleus.

### RNA extraction, cDNA synthesis, and quantitative real-time polymerase chain reaction

Total RNA was extracted from transfected and non-transfected cells using the TRIzol Universal Reagent kit (TIANGEN, Beijing, China) as per the manufacturer’s instructions. 1 µg of RNA was reverse transcribed into cDNA using the First-Strand cDNA Synthesis Kit (TaKaRa, Tokyo, Japan). Quantitative real-time PCR (RT-qPCR) was performed using a Bio-Rad RT-PCR system (Hercules, CA, USA) with SYBR Green (TIANGEN, Beijing, China) to assess the expression levels of *SREBP1, SREBP2, LPL, SCD1, CPT1, DGAT2, STAR, and CYP17A1,* normalized to *GAPDH*. Each sample was analyzed in triplicate. Primer sequences are provided in Table 1.

### Western blot analysis

For protein expression analysis, GCs were cultured in six-well plates and transfected with either the overexpression plasmid or siRNA for 48 h. Total protein was extracted using a protein extraction kit (BestBio Biotech Co., Ltd., Shanghai, China), and protein concentrations were determined using the BCA protein quantification kit (BestBio). The primary antibodies used in this work including anti-NPC2 (ABclonal, A5413, dilution1:1000), anti-CYP17A1 (Zenbio, P05093, dilution 1:1000), anti-β-Tubulin (Zenbio, 200608, dilution 1:5000), anti-StAR (Abclonal, A1035, dilution 1:1000). anti-StAR (Abclonal, A1035, dilution 1:1000), anti-SCD1 (Zenbio, R25675, dilution 1:1000), anti-LPL (Zenbio, R381844, dilution 1:1000), The β-Tubulin was used as a loading control. Then the western blot analysis and immunofluorescence imaging were performed according to previously described methods(Cui et al., 2020). Thereafter, fluorescence images were captured using a fluorescence microscope (Olympus, Melville, NY, USA), and band intensities from Western blots were quantified using ImageJ software.

### Enzyme-linked immunosorbent assay (ELISA) and measurement of intracellular triglyceride and cholesterol concentrations

To determine steroid hormone concentrations, culture medium supernatants were collected 48 h post-transfection, and estradiol (E₂) and progesterone (P₄) levels were measured using commercial chicken ELISA kits (Jianglai Bio, Shanghai, China) following the manufacturer’s protocol. In addition, total cellular protein was extracted, and total cholesterol (TC) and triglyceride (TG) levels were quantified using chicken TC and TG ELISA kits (Nanjing Jiancheng Bioengineering Institute, Nanjing, China).

### *Statistical Analysis*

All data were subjected to statistical analysis using SPSS 27.0 statistical software (SPSS, Inc., Chicago, Illinois, USA). There were three biological replicates for each experiment. Comparisons between two groups were conducted using an unpaired Student’s t-test, The experimental data were initially assessed for normal distribution, and based on this, a one-way analysis of variance (ANOVA) was conducted, including a homogeneity test of variance. One-way ANOVA, combined with Duncan’s multiple range test was used for comparative analysis of the groups, with significant level set at *P* < 0.05 (*) and *P* < 0.01 (**).

## Results

### Identification of chicken granulosa cells and detection of transfection efficiency

**Fig. 1:**
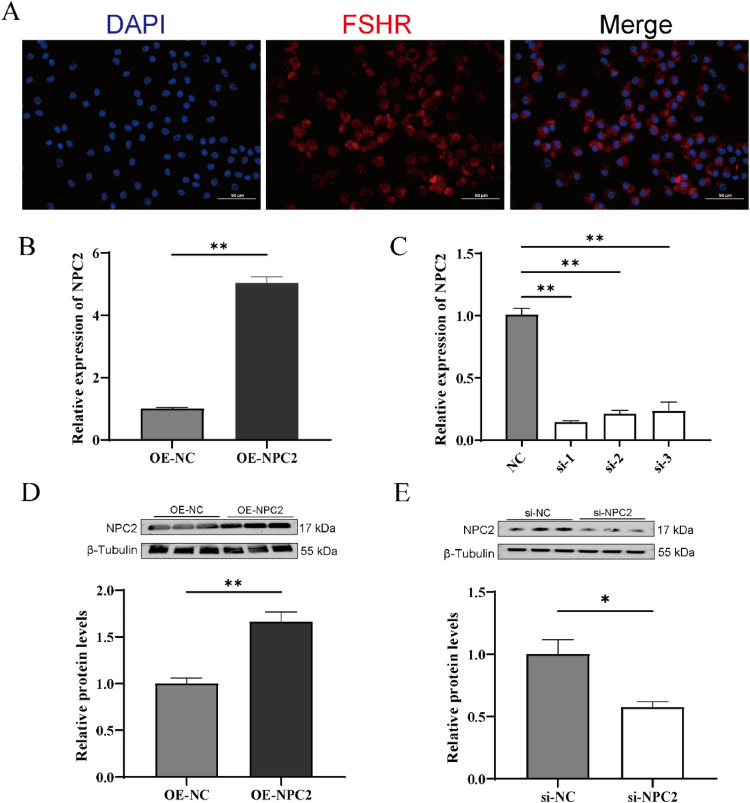
Identification of chicken granulosa cells and detection of transfection efficiency. (A) Immunofluorescence staining of FSHR in the chicken granulosa cells. (B, C) Relative expression of *NPC2* after transfection with *NPC2* overexpression vector or siRNAs. (D, E) Relative protein level of *NPC2* after transfection with *NPC2* overexpression vector or siRNAs. Each treatment had at least three replicates and the results are expressed as mean ± SEM. *P < 0.05; **P < 0.01.

FSHR protein is widely recognized as a marker for identifying granulosa cells due to its high specificity and elevated expression in these cells (Simoni et al., 1997). The results from the immunofluorescence staining confirmed strong FSHR expression in all cells, indicating that the isolated primary granulosa cells were of high purity and viability, making them suitable for subsequent experiments (Fig. 1A). qPCR analysis revealed that *NPC2* mRNA levels increased by fivefold in granulosa cells following transfection with the OE-NPC2 plasmid (Fig. 1B). Among the three siRNAs targeting *NPC2*, si-NPC2-1 exhibited the highest interference efficiency. Consequently, si-NPC2-1 was selected for further experiments, reducing *NPC2* mRNA levels in granulosa cells by 15 % (Fig. 1C). In addition, *NPC2* protein expression levels were consistent with the qRT-PCR findings (Fig. 1D and 1E).

### NPC2 inhibits lipid deposition in the chicken GC

**Fig. 2:**
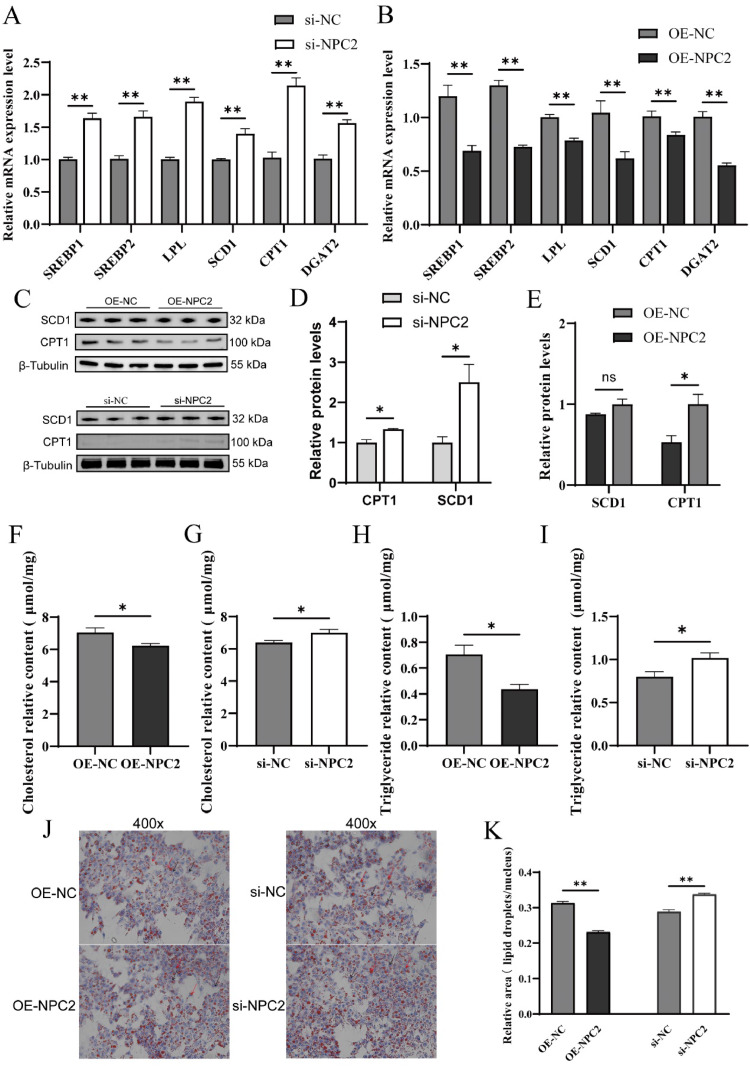
*NPC2* inhibits lipid deposition of chicken granulosa cells (GC). (A, B) The mRNA expression of lipid metabolism-related genes (*SREBP1, SREBP2, LPL, SCD1, CPT1*, and *DGAT2*) was determined after transfection with *NPC2* overexpression vector or siRNAs. (C, D) Protein expression levels of SCD1 and CPT1 after transfection. (E-H) effect on the content of total TC and TG (TC: cholesterol, TG: triglyceride) after transfection. (I, J) Area of lipid droplets relative to nucleus in the GC after transfection. Black arrows indicate the nucleus, red arrows indicate lipid droplets. Each treatment had at least three replicates and results are expressed as mean ± SEM. ^ns^*P* ≥ 0.05; * *P* < 0.05; **P < 0.01.

Following transfection, we assessed the mRNA expression levels of key genes involved in lipid metabolism, including *SREBP1, SREBP2, LPL, SCD1, CPT1*, and *DGAT2. NPC2* knockdown significantly upregulated the mRNA expression of these genes (*P* < 0.01) (Fig. 2A), whereas *NPC2* overexpression led to a marked reduction in their relative mRNA levels (*P* < 0.01) (Fig. 2B). The protein expression trends for SCD1 and CPT1 were consistent with the mRNA results (*P* < 0.05) (Fig. 2C–2E). However, SCD1 protein expression remained unchanged in the OE-NPC2 group, suggesting that *NPC2* may play an inhibitory role in lipid synthesis in granulosa cells. To further investigate the role of *NPC2* in lipid metabolism, we quantified intracellular total cholesterol (TC) and triglyceride (TG) levels using ELISA. *NPC2* overexpression significantly decreased TC and TG content in granulosa cells (*P* < 0.05) (Fig. 2F and 2H), while *NPC2* knockdown resulted in a notable increase in these lipid metabolites (*P* < 0.05) (Fig. 2G and 2I). These findings were further supported by Oil Red O staining, which showed that the area of lipid droplets relative to the nucleus in the granulosa cells was significantly decreased in the NPC2 overexpression (OE-NPC2) group and significantly increased in the siRNA-NPC2 group compared to the control (*P* < 0.01) (Fig. 2K). Collectively, these results indicate that *NPC2* suppresses lipid deposition in chicken granulosa cells.

### NPC2 promotes steroid hormone biosynthesis in chicken GC

**Fig. 3:**
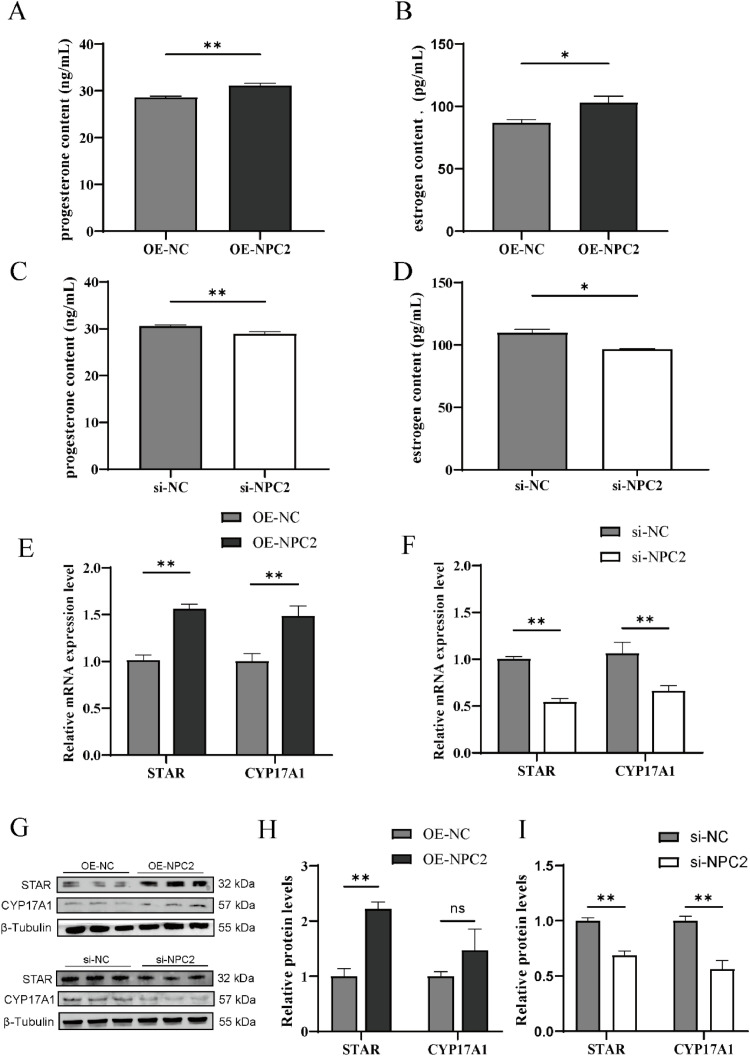
*NPC2* promotes and progesterone biosynthesis in GC. (A, B) Estrogen contents calculated using microplate reader after transfection with *NPC2* overexpression vectors or siRNAs. (C, D) Progesterone contents calculated using microplate reader after transfection. (E, F) Relative expression of *STAR* and *CYP17A1* after transfection with *NPC2* overexpression vectors or siRNAs. (G, H) Protein expression of STAR and CYP17A1 after transfection. Each treatment had at least three replicates and results are expressed as mean ± SEM. ^ns^*P* ≥ 0.05; * *P* < 0.05; **P < 0.01.

The levels of estradiol (E_2_) and progesterone (P_4_) were assessed in the cell culture supernatant. As shown in Fig. 3A–3D, *NPC2* overexpression significantly increased E_2_ and P_4_ secretion (*P* < 0.05), whereas *NPC2* knockdown led to a notable reduction in their levels (*P* < 0.05).

To explore the underlying molecular mechanisms, we examined the mRNA expression of *CYP17A1* and *STAR*, key genes involved in steroid hormone biosynthesis. The results showed that *NPC2* overexpression significantly upregulated their expression (*P* < 0.01) (Fig. 3E), while *NPC2* knockdown resulted in their downregulation (*P* < 0.01) (Fig. 3F). Western blot analysis confirmed that the protein expression patterns of STAR and CYP17A1 were consistent with the mRNA findings (*P* < 0.01) (Fig. 3G–3I). However, CYP17A1 protein expression remained unchanged in the OE-NPC2 group. These findings suggest that *NPC2* plays a crucial role in promoting steroid hormone synthesis while inhibiting lipid accumulation in chicken granulosa cells.

## Discussion

Lipids in poultry egg yolk are primarily derived from dietary sources or synthesized through the de novo lipogenesis (DNL) pathway in animal tissues, with approximately 90-95 % of this process occurring in the liver (Huang et al., 2022). However, recent studies have shown that granulosa cells (GCs) in geese are capable of synthesizing triglycerides (TG) through de novo lipid synthesis. Moreover, the accumulation of lipid droplets in GCs increases as follicle size expands (Wen et al., 2019; Gao et al., 2019).

Recent findings indicate that under lipid-deficient conditions, *de novo* lipid synthesis and lipid uptake are upregulated, and transcription factors such as *SREBF1/SREBP-1a* and *SREBF1/SREBP-1c* enhance the expression of *NPC2* (Geng and Guo, 2024). In our study, we observed that *NPC2* overexpression and silencing respectively downregulated and upregulated the expression of key lipid metabolism-related genes, including *SREBP1, SREBP2, LPL*, and *CPT1*. Previous studies have demonstrated that SREBP1/SREBP2 is a crucial pathway in lipid metabolism that suppresses lipid transport while promoting lipid accumulation in chicken follicular GCs (Prasad et al., 2022). In addition, previous research found that silencing *LPL* led to a reduction in lipid droplet formation in granulosa cells and a decrease in *SREBP1* expression (Cui et al., 2022). These findings, in conjunction with our current results, confirm the role of *NPC2* in regulating lipid metabolism in chicken GCs. In this study, we also found that *DGAT2* expression was increased upon *NPC2* knockdown and decreased with *NPC2* overexpression. Furthermore, the ratio of lipid droplet area to total cell area, as well as the cellular levels of total cholesterol (TC) and triglycerides (TG), followed the same pattern. Previous research has shown that lipid components such as TC and TG in chicken egg yolk serve as an energy source for GC growth and development (Ma et al., 2020; Yuan et al., 2021). Among these, *NPC2* is a key regulator of cholesterol transport, while *DGAT2* plays a critical role in TG synthesis (Hung et al., 2017; Chitraju et al., 2019). These results further suggest that *NPC2* influences follicular development by modulating lipid metabolism in chicken GCs.

*CYP17A1* is a well-established rate-limiting enzyme in androgen biosynthesis, catalyzing key steps such as the conversion of pregnenolone to dehydroepiandrosterone (DHEA) and progesterone to 17α-hydroxyprogesterone. These intermediates are further processed to produce androstenedione and estradiol (E_2_), which play central roles in maintaining sex hormone homeostasis (Tsutsui et al., 2013). As a pivotal regulator of steroid hormone synthesis, *CYP17A1* significantly influences reproductive physiology and is implicated in the pathogenesis of hormone-related disorders. For example, elevated ovarian expression of *CYP17A1* has been associated with increase androgen and metabolic disturbances in polycystic ovary syndrome (PCOS), which contributes to hyperandrogenism, disrupted folliculogenesis, and increased risk of infertility (Rosenfield and Ehrmann, 2016; Choi et al., 2024).

Meanwhile, *STAR* facilitates the transport of cholesterol into mitochondria, where it serves as a precursor for steroid hormone synthesis (Stocco, 2001; Johnson and Bridgham, 2001). Studies have shown that knockout of the *STAR* gene in mice leads to defects in mitochondrial structure. Interestingly, overexpression of *STAR* in STARKO1 cells exacerbates these defects rather than reversing them (Galano et al., 2021). Furthermore, knockdown of *STAR* results in increased cellular lipid droplet density, along with a significant accumulation of cholesterol esters and diglycerides (Galano and Papadopoulos, 2022). These findings suggest that *STAR* regulates steroid hormone synthesis by modulating cholesterol transport to mitochondria, a critical step for initiating steroidogenesis. In this present study, *NPC2* overexpression upregulated *STAR* and *CYP17A1* expression, whereas *NPC2* knockdown led to their suppression. However, the protein expression levels of CYP17A1 and SCD1 remained unchanged in the OE-NPC2 group. This observation is consistent with previous reports indicating that both SCD1 and CYP17A1 exhibit slow protein turnover, and transient reductions in mRNA expression may not result in immediate changes at the protein level (Ascenzi et al., 2021; Li et al., 2025). This indicates that *NPC2* is involved in regulating steroid hormone synthesis in chicken GCs.

**Fig. 4:**
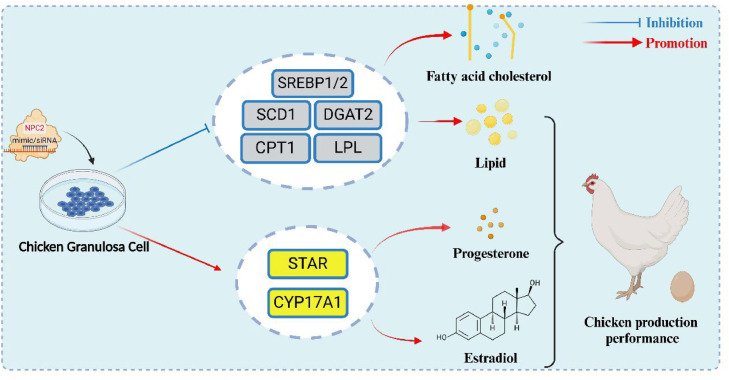
Schematic Diagram of the *NPC2* Pathway in Lipid Deposition and Steroid Hormone Secretion in Chicken Granulosa Cells. The *NPC2* plays a dual role in chicken granulosa cells by regulating lipid deposition and steroid hormone secretion. It inhibits *SREBP1/2*, thereby reducing lipid biosynthesis and triglyceride accumulation while promoting *STAR* and *CYP17A1*, thereby enhancing progesterone and estradiol production. Hence, this balance supports follicular development and ovarian function.

Recent studies have demonstrated that *NPC2* in the mouse ovary facilitates the production of E_2_ and progesterone (P_4_) using cholesterol as a substrate (Busso et al., 2010). In addition, *SCD1*, a key gene involved in adipogenesis, has been shown to influence P_4_ synthesis in goose GCs when its expression is modulated via interference and overexpression vectors (Yuan et al., 2022). Our findings in chicken GCs support this relationship, as *SCD1* expression was downregulated upon *NPC2* knockdown and upregulated upon *NPC2* overexpression. Correspondingly, E_2_ and P_4_ levels increased following *NPC2* knockdown and decreased with *NPC2* overexpression, further highlighting the link between lipid metabolism and steroid hormone biosynthesis in chicken GCs (Fig. 4). These results suggest that modulating *NPC2* expression could offer a novel molecular target for enhancing reproductive performance in laying hens. Therefore, selective breeding programs could prioritize hens with favorable *NPC2* expression profiles associated with improved follicular lipid metabolism and steroid hormone output. In addition, dietary strategies that upregulate *NPC2* such as supplementation with cholesterol precursors or activators of *SREBP* pathways may optimize granulosa cell function, and improve yolk precursor biosynthesis. Further investigation into feed additives or nutrigenomic interventions targeting *NPC2* could thus lead to measurable gains in egg-laying efficiency.

## Conclusions

In conclusion, our findings demonstrate that *NPC2* suppresses lipid accumulation while promoting steroid hormone synthesis in chicken granulosa cells. This study provides new insights into the molecular mechanisms underlying lipid metabolism and steroid hormone production in poultry, contributing to a better understanding of follicular development and ovarian function in laying hens. Furthermore, the regulatory role of *NPC2* in lipid and hormone metabolism offers a promising target for improving reproductive efficiency in commercial poultry. Its modulation through genetic selection or nutritional intervention may serve as a practical strategy to enhance follicular development, optimize hormone profiles, and ultimately boost egg production in laying hens.

## Declaration of competing interest

The authors declare that they have no known competing financial interests or personal relationships that could have appeared to influence the work reported in this paper.
